# Antioxidant Activities and Phytochemical Study of Leaf Extracts from 18 Indigenous Tree Species in Taiwan

**DOI:** 10.1155/2012/215959

**Published:** 2012-02-02

**Authors:** Shang-Tse Ho, Yu-Tang Tung, Yong-Long Chen, Ying-Ying Zhao, Min-Jay Chung, Jyh-Horng Wu

**Affiliations:** ^1^Department of Forestry, National Chung Hsing University, Taichung 402, Taiwan; ^2^Department of Life Sciences, National Chung Hsing University, Taichung 402, Taiwan; ^3^The Experimental Forest, National Taiwan University, Taipei 106, Taiwan

## Abstract

The objective of this study is to assess antioxidant activities of methanolic extracts from the leaves of 18 indigenous tree species in Taiwan. Results revealed that, among 18 species, *Acer oliverianum* exhibited the best free radical scavenging activities. The IC_50_ values were 5.8 and 11.8 *μ*g/mL on DPPH radical and superoxide radical scavenging activities, respectively. In addition, *A. oliverianum* also exhibited the strongest ferrous ion chelating activity. Based on a bioactivity-guided isolation principle, the resulting methanolic crude extracts of *A. oliverianum* leaves were fractionated to yield soluble fractions of hexane, EtOAc, BuOH, and water. Of these, the EtOAc fraction had the best antioxidant activity. Furthermore, 8 specific phytochemicals were isolated and identified from the EtOAc fraction. Among them, 1,2,3,4,6-*O*-penta-galloyl-**β**-D-glucopyranose had the best free radical scavenging activity. These results demonstrate that methanolic extracts and their derived phytochemicals of *A. oliverianum* leaves have excellent antioxidant activities and thus they have great potential as sources for natural health products.

## 1. Introduction

Molecular and cellular damage due to reactive oxygen species (ROS) is widely believed to be the major cause of aging, neural disorders, diabetes, atherosclerosis, inflammatory injury, cancer, and cardiovascular disease [1]. Thus, a potential scavenger of ROS may serve as a possible preventive intervention for free-radical-mediated diseases [2]. Plants are potential sources of natural antioxidants. In the past few years, the antioxidant properties of plants have been extensively studied [3, 4]. Among the various medicinal and culinary plants, some endemic species are of particular interest because they may be used for preparations containing phytochemicals with significant antioxidant activities and health benefits [5]. Therefore, the intake of natural antioxidants from plants has been associated with low incidence of cancer, cardiovascular disease, diabetes, and other diseases associated with aging [6].

Taiwan is on the boundary of the tropics and subtropics, and although the island covers only a small area, the environment of Taiwan is diversified, possessing unique indigenous tree species in this island. However, to the best of our knowledge there is no prior report on antioxidative phytochemicals of leaf extracts of indigenous tree species in Taiwan. Thus, in this study, a number of *in vitro* assays were performed to evaluate the antioxidant activities of methanolic extracts from the leaves of 18 indigenous tree species. In addition, the characteristics of bioactive phytochemicals were also addressed in this study.

## 2. Materials and Methods

### 2.1. Extraction and Fractionation of 18 Indigenous Tree Species

The leaves of 18 indigenous tree species in Taiwan were collected at the end of May 2009 from the Hui-Sun Forest Station of National Chung Hsing University (NCHU) in Nantou County. All voucher specimens including *Acacia confusa* Merr. (Lu 0069), *Acer oliverianum* Pax. var. *nakaharai* (Peng 0001), *Calocedrus formosana* Florin (Tsai et al. S.N.), *Cunninghamia lanceolata* Hook. var. *konishii *(Lin 0002), *Cunninghamia lanceolata* Hook. var. *lanceolata* (Hsu 0013), *Cryptomeria japonica* D. Don (Wang 0007), *Evodia lepta* Merr. (Wu 0030), *Fraxinus formosana* Hay. (Tseng 2706), *Helicia rengetiensis *Masamune (Tseng 2588), *Liquidambar formosana* Hance (Liu 0007), *Machilus kusanoi* Hay. (Chen 0025), *Neolitsea konishii* Kaneh. et Sasaki (Ou et al. 7355), *Pinus morrisonicola* Hay. (Chiu 4127), *Pinus taiwanensis* Hay. (Ou et al. 9007), *Styrax formosana* Matsum. (Lin 0018), *Taiwania cryptomerioides* Hay. (Liu S.N.), *Viburnum luzonicum* Rolfe (Chen 0049), and *Zelkova serrata* Makino (Lu et Ou 0964) were deposited at the herbarium of the Department of Forestry, NCHU. The species were identified by Dr. Yen-Hsueh Tseng (Department of Forestry, NCHU). The samples were cleaned with tap water and dried. Then they were extracted with methanol by ultrasound-assisted extraction for 30 min at room temperature two times. The leaf extracts of 18 indigenous tree species were decanted, filtered under vacuum, concentrated in a rotary evaporator, and then lyophilized. Furthermore, the resulting methanolic crude extracts of *A. oliverianum* were fractionated successively with *n*-hexane, ethyl acetate (EtOAc), *n*-butanol (BuOH), and water to yield soluble fractions of hexane, EtOAc, BuOH, and water. All extracts were stored in an airtight container at −40°C prior to further analysis.

### 2.2. 1,1-Diphenyl-2-picrylhydrazyl Assay (DPPH Assay)

The DPPH radical scavenging activity of the test extracts was examined according to the method reported by Tung et al. [7]. Ten microliters of the test samples in methanol was mixed with 200 *μ*L of 0.1 mM DPPH-ethanol solution and 90 *μ*L of 50 mM Tris-HCl buffer (pH 7.4). Methanol (10 *μ*L) alone was used as the control of this experiment. After 30 min of incubation at room temperature, the reduction in DPPH radicals was measured by reading the absorbance at 517 nm. (+)-Catechin was used as the positive control. The inhibition ratio was calculated using the following equation:

(1) $$\begin{array}{cc} & \text{\%}\;\text{inhibition}\\ & =\frac{\;\text{absorbance}\;\text{of}\;\text{control}-\text{absorbance}\;\text{of}\;\text{test}\;\text{sample}}{\text{absorbance}\;\text{of}\;\text{control}}\\ & \;\times 100. \end{array}$$

### 2.3. Superoxide Radical Scavenging Assay (NBT Assay)

Measurement of superoxide radical scavenging activity was carried out according to the method of Ho et al. [8]. First, 20 *μ*L of 15 mM Na_2_EDTA in buffer (50 mM KH_2 _PO_4_/KOH, pH 7.4), 50 *μ*L of 0.6 mM NBT in buffer, 30 *μ*L of 3 mM hypoxanthine in 50 mM KOH, 5 *μ*L of test samples in methanol, and 145 *μ*L of buffer were mixed in 96-well microplates. The reaction was started by adding 50 *μ*L of xanthine oxidase solution in buffer (1 unit in 10 mL buffer) to the mixture. The reaction mixture was incubated at room temperature, and the absorbance at 570 nm was determined every 1 min up to 9 min using a plate reader (Labsystems Multiskan MS, Finland). The control was 5 *μ*L of methanol instead of the sample solution. (+)-Catechin was used as the positive control. The inhibition ratio was calculated using the following equation:

(2) $$\begin{array}{cc} & \text{\%}\;\text{Inhibition}\\ & =\frac{\text{rate}\;\text{of}\;\text{control}\;\text{reaction}-\text{rate}\;\text{of}\;\text{sample}\;\text{reaction}}{\text{rate}\;\text{of}\;\text{control}\;\text{reaction}}\\ & \;\times 100. \end{array}\;$$

### 2.4. Ferrous Ion Chelating Assay

The ferrous ion chelating potential of the test samples was evaluated according to the method of Tung et al. [9]. Briefly, 200 *μ*L of the test samples in methanol and 740 *μ*L methanol were added to 20 *μ*L of 2 mM FeCl_2_. The reaction was initiated by adding 40 *μ*L of 5 mM ferrozine. The mixture was shaken vigorously and rested at room temperature for 10 min. Absorbance of the solution was then measured at 562 nm. EDTA was used as a positive control. The percent of inhibition of Fe^2+^-ferrozine complex formation was calculated according to the following equation:

(3) $$\begin{array}{cc} & \text{\%}\;\text{inhibition}\\ & =\frac{\text{absorbance}\;\text{of}\;\text{control}-\text{absorbance}\;\text{of}\;\text{sample}}{\text{absorbance}\;\text{of}\;\text{control}}\\ & \;\times 100. \end{array}\;$$

### 2.5. Determination of Total Phenolics of Different Indigenous Species in Taiwan

Total phenolic contents were determined according to the Folin-Ciocalteu method [10], using gallic acid as the standard. The test samples (5 mg) were dissolved in 5 mL of methanol/water (50 : 50, v/v). The extract solution (500 *μ*L) was mixed with 500 *μ*L of 50% Folin-Ciocalteu reagent. The mixture was kept for 5 min, which was followed by the addition of 1.0 mL of 20% Na_2_CO_3_. After 10 min of incubation at room temperature, the mixture was centrifuged for 8 min (12000 g), and the absorbance of the supernatant was measured at 730 nm. The total phenolic content was expressed as gallic acid equivalents (GAE) in milligrams per gram sample.

### 2.6. Isolation and Identification of Bioactive Phytocompounds

Based on bioactivity-guide isolation principle, the EtOAc soluble fraction from the *A. oliverianum* had an excellent antioxidant activity, thus it was loaded into a chromatography column (Geduran Si-60, Merck, Darmstadt, Germany) and eluted with gradient EtOAc*/ n*-hexane and MeOH/EtOAc solvent systems, and 9 subfractions (EA1–9) were obtained. The antioxidative phytochemicals from the EA5, EA6, and EA7 were separated and purified by semipreparative HPLC using a PU-2080 pump (Jasco, Japan) equipped with a MD-2010 multiwavelength detector (Jasco, Japan) and a 250 × 10.0 mm i.d., 5-*μ*m Ascentis RP-amide column (Supelco, Bellefonte, USA). The mobile phase was solvent *A*, 100% MeOH; and solvent *B*, ultrapure water. Elution condition was 0–24 min of 4–100%  *A* to *B* (linear gradient) at a flow rate of 4 mL/min for isolation of EA5. On the other hand, the elution condition was 0–5 min of 40–64%  *A* to *B* (linear gradient), 5–11 min of 64%  *A* to *B*, 11–37 min of 64–85%  *A* to *B* (linear gradient), and 37–42 min of 85–100%  *A* to *B* (linear gradient) at a flow rate of 4 mL/min for isolation of EA6 and EA7. The structures of compounds **1**–**8** were determined by MS (Finnigan MAT-95S, Germany) and ^1^H NMR (Varian Unity Inova-600, USA), and all spectral data were consistent with the published literature [11–17].

### 2.7. Statistical Analysis

The significance of difference was calculated by Scheffe's test, and results with *P* < 0.05 were considered statistically significant. Comparisons of total phenolic contents and various antioxidant activities were carried out using Pearson's correlation test.

## 3. Results and Discussion

### 3.1. The Yields of Methanolic Extracts of 18 Indigenous Tree Species in Taiwan

The leaves of 18 indigenous tree species in Taiwan yielded from 6.6 to 27.3% (w/w) methanolic extracts based on dry weight. Of these, the yields of 10 species, including *S. formosana *(27.3%), *V. luzonicum* (21.4%), *F. formosana* (16.7%),* E. lepta* (16.1%), *L. formosana *(15.7%),* P. morrisonicola *(15.0%), *C. lanceolata *var.* konishii* (12.5%), *H. rengetiensis *(12.3%), *A. oliverianum* (10.5%), and *A. confusa* (10.5%), were higher than 10%. In addition, the yields among different species are very different, for example, the yield *of S. formosana* (27.3%) is fourfold the *N. konishii *(6.6%).

### 3.2. Antioxidant Activities of Methanolic Extracts of 18 Indigenous Tree Species in Taiwan

As for the inhibitory effects of the leaf extracts from 18 indigenous species in Taiwan on DPPH radicals, data in Table 1 shows that most extracts revealed a good scavenging activity for DPPH radicals. The IC_50_ values (the concentration required to inhibit radical formation by 50%) of crude extracts increased in the following order: *A. oliverianum *(5.8 *μ*g/mL) > *Z. serrata* (9.7 *μ*g/mL) > *A. confusa* (12.9 *μ*g/mL) *> L. formosana* (14.9 *μ*g/mL) >* V. luzonicum* (15.3 *μ*g/mL) > *F. formosana* (24.2 *μ*g/mL) > *N. konishii* (25.1 *μ*g/mL) > *P. morrisonicola* (25.9 *μ*g/mL) >* H. rengetiensis* (31.6 *μ*g/mL) >* C. formosana* (33.3 *μ*g/mL) > *T. cryptomerioides* (35.9 *μ*g/mL) > *C. lanceolata* var.* konishii* (37.7 *μ*g/mL) > *S. formosana* (38.1 *μ*g/mL) >* P. taiwanensis* (38.3 *μ*g/mL) > *M. kusanoi* (42.2 *μ*g/mL) > *E. lepta* (76.7 *μ*g/mL) > *C. japonica* (98.3 *μ*g/mL) >* C. lanceolata *var.* lanceolata* (98.9 *μ*g/mL). Of these, the IC_50_ values of four species, including *A. oliverianum*, *Z. serrata*, *A. confusa,* and *L. formosana,* were lower than 15 *μ*g/mL. In comparison with a well-known antioxidant, (+)-catechin (IC_50_ = 2.6 *μ*g/mL), the crude extracts of the trees mentioned above exhibited a good DPPH radical-scavenging activity. Additionally, the crude extract of green tea showed an excellent inhibitory activity against DPPH radicals with IC_50 _value of 5 *μ*g/mL [18]. Comparison of the results indicates that the leaf extracts of* A. oliverianum* would be an excellent source of natural antioxidants and merit further investigation.

Furthermore, superoxide radical scavenging activity of the test samples from different indigenous species in Taiwan was determined by the hypoxanthine-xanthine oxidase system. Table 1 shows the superoxide radical scavenging activity of various methanolic extracts compared with (+)-catechin. The inhibitory activity of 18 species was observed in a dose-dependant manner, and the leaf extracts of *A. oliverianum* exhibited the highest superoxide radical scavenging activity among all species. The IC_50_ values of (+)-catechin,* A. confusa*, *A. oliverianum*, *C. formosana*, *C. japonica*,* C. lanceolata *var.* lanceolata*,* C. lanceolata* var.* konishii*,* E. lepta*, *F. formosana, H. rengetiensis*, *L. formosana, M. kusanoi*, *N. konishii*, *P. morrisonicola*,* P. taiwanensis, S. formosana*, *T. cryptomerioides*,* V. luzonicum, *and *Z. serrata* were 8.8, 22.3, 11.8, 35.8, >100, >100, 44.2, >100, 44.4, 42.6, 30.9, >100, 37.3, 26.1, 38.7, 21.9, 36.0, 24.7, and 23.6 *μ*g/mL, respectively. This result was similar to that of DPPH assay for all the species, *A. oliverianum* exhibited the strongest superoxide radical scavenging activity.

On the other hand, the chelating effect of the test sample on ferrous ions is also shown in Table 1. Results revealed that IC_50_ values of chelating effect for various leaf extracts were as follows: *A. oliverianum* (88.1 *μ*g/mL) > *L. formosana* (123.1 *μ*g/mL) > *M. kusanoi* (185.3 *μ*g/mL) > *N. konishii* (251.6 *μ*g/mL) > *C. lanceolata *var.* lanceolata* (297.9 *μ*g/mL) > *V. luzonicum* (316.3 *μ*g/mL) > *A. confusa* (356.7 *μ*g/mL) > *Z. serrata* (397.0 *μ*g/mL) >* C. lanceolata* var.* konishii* (439.5 *μ*g/mL) > *E. lepta* (581.1 *μ*g/mL) > *C. formosana* (607.9 *μ*g/mL) > *F. formosana* (654.5 *μ*g/mL) > *T. cryptomerioides* (795.3 *μ*g/mL) > *P. morrisonicola* (885.3 *μ*g/mL) > *S. formosana* (907.5 *μ*g/mL) >* C. japonica* (>1000 *μ*g/mL) = *H. rengetiensis* (>1000 *μ*g/mL) = *P. taiwanensis* (>1000 *μ*g/mL). Comparison of the aforementioned results obtained from free radical scavenging activities indicated that the ferrous ion chelating effect of methanolic extracts did not correlate with the results from DPPH and NBT assays used to estimate antioxidant activities. This discrepancy in the antioxidant assays may be due to different mechanisms involved in antioxidant assays. Furthermore, our findings are also in agreement with the results reported by Chua et al. [19] and Tung et al. [20]. However, among 18 species, the leaf extracts of *A. oliverianum* still exhibited better ferrous ion chelating effect than others.

### 3.3. Total Phenolics of Methanolic Extracts of 18 Indigenous Tree Species in Taiwan

It is well known that plant phenolics (e.g., flavonoids and proanthocyanidins) are generally highly effective free radical scavengers and antioxidants. From the estimation of phenolic contents it can be observed that the polyphenolics and antioxidant activities are a combined measure of the quality and quantity of antioxidants. Table 1 shows that the contents of total phenolics in crude extracts were determined spectrometrically according to the Folin-Ciocateu method and calculated as gallic acid equivalents (GAE). Accordingly, total phenolic contents of different indigenous species in Taiwan were in decreasing order: *A. oliverianum* (311.7 mg GAE/g) > *V. luzonicum* (210.9 mg GAE/g) > *Z. serrata* (200.9 mg GAE/g) > *A. confusa* (190.2 mg GAE/g) > *F. formosana* (183.9 mg GAE/g) > *S. formosana* (151.5 mg GAE/g) > *C. formosana* (150.3 mg GAE/g) > *L. formosana* (147.4 mg GAE/g) > *P. taiwanensis* (120.1 mg GAE/g) > *P. morrisonicola* (108.9 mg GAE/g) > *C. lanceolata* var.* konishii* (105.2 mg GAE/g) > *N. konishii* (100.0 mg GAE/g) > *E. lepta* (72.8 mg GAE/g) > *T. cryptomerioides* (70.1 mg GAE/g) > *H. rengetiensis* (63.4 mg GAE/g) > *C. lanceolata *var.* lanceolata* (61.6 mg GAE/g) > *M. kusanoi* (54.7 mg GAE/g) > *C. japonica* (18.9 mg GAE/g). This result revealed that *A. oliverianum*, *V. luzonicum*, *Z. serrata,* and *A. confusa* leaf extracts showed the higher phenolic contents, which correlated with the results of free radical scavenging activities. Previous report also found that the antioxidant effects of phenolics are strongly dependent on the choice of raw materials because the antioxidant activities differ between different phenolic constituents [21]. On the basis of the results obtained, effective antioxidants from methanolic extracts of *A. oliverianum* leaves can be obtained to separate and purify.

### 3.4. Correlation Coefficients among DPPH Radical Scavenging Activity, Superoxide Radical Scavenging Activity, Ferrous Ion Chelating Ability, and Total Phenolic Contents in Extracts

Phenolic compounds are very important plant constituents because of their scavenging ability due to their hydroxyl groups. Thus, the correlation between the total phenolic contents and antioxidant activities has been widely studied in different plants or foodstuffs [22–25]. In this study, correlation coefficients for total phenolic contents with the DPPH, NBT, and ferrous ion chelating assays are shown in Table 2. A linear relationship was also established between total phenolic content and antioxidant activities and it was observed that antioxidant activities increased proportionally to the phenolic contents. These results showed that strong correlations were obtained between total phenolic contents and DPPH assay, NBT assay, and ferrous ion chelating assay, with *R* ^2^ values of 0.71 (*P* < 0.01), 0.72 (*P* < 0.01), and 0.54 (*P* < 0.05), respectively. Furthermore, antioxidant activities of extracts are correlated to their phenolic contents, and it is proposed that phenolic compounds from the methanolic extracts of *A. oliverianum* leaves may play an important role in antioxidant activities. Thus, the therapeutic properties of *A. oliverianum* may be attributed to the phenolic compounds.

### 3.5. Isolation and Identification of Bioactive Phytocompounds

**Figure 1:**
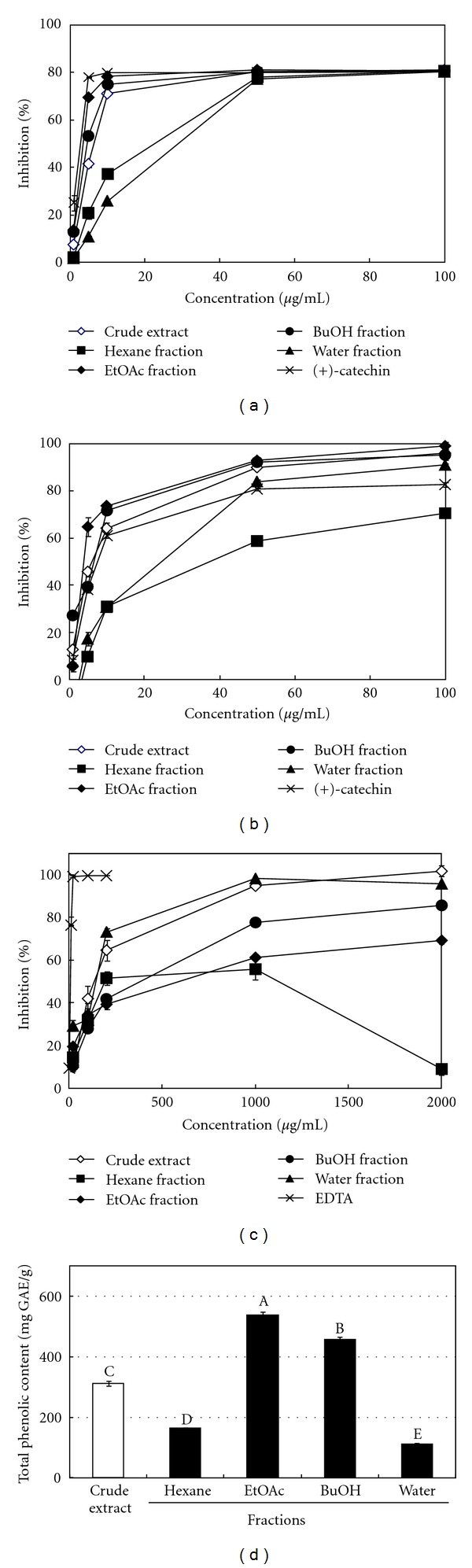
Antioxidant activities of methanolic extracts and their derived soluble fractions from the leaves of *A. oliverianum*. (a) DPPH radical scavenging activity. (b) Superoxide radical scavenging activity. (c) Ferrous ion chelating ability. (d) Total phenolic contents. Results are mean ± SD (*n* = 3). The bars marked by different letters are significantly different at the level of *P* < 0.05 according to the Scheffe's test.

**Figure 2:**
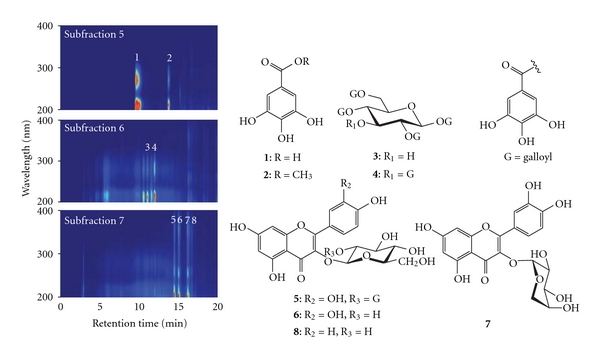
HPLC chromatograms and the major phytochemicals of EA5, EA6, and EA7 from the leaves of *A. oliverianum*. (**1**) Gallic acid, (**2**) gallic acid methyl ester, (**3**) 1,2,4,6-*O*-tetra-galloyl-*β*-d-glucopyranoside, (**4**) 1,2,3,4,6-*O*-penta-galloyl-*β*-d-glucopyranoside, (**5**) quercetin 3-*O*-*β*-d-(2′′-galloyl)-glucopyranoside, (**6**) quercetin 3-*O*-*β*-d-glucopyranoside, (**7**) quercetin 3-*O*-*α*-l-arabinopyranoside, and (**8**) kaempferol 3-*O*-*β*-d-glucopyranoside.

Based on bioactivity-guided isolation principle, the resulting methanolic crude extracts of *A. oliverianum* leaves were fractionated successively with *n*-hexane, ethyl acetate (EtOAc), *n*-butanol (BuOH), and water to yield soluble fractions of hexane, EtOAc, BuOH, and water. As shown in Figure 1, the DPPH radical and superoxide radical scavenging activities of methanolic extracts and their derived soluble fractions from *A. oliverianum* leaves increased with increasing the concentration of the test sample. The IC_50_ values of crude extract, hexane fraction, EtOAc fraction, BuOH fraction, and water fraction of *A. oliverianum* leaves were 5.8, 16.3, 3.2, 4.1, and 20.3 *μ*g/mL on DPPH assay (Figure 1(a)); 11.8, 30.4, 3.9, 6.1, and 25.9 *μ*g/mL on NBT assay (Figure 1(b)); 88.1, 191.3, 609.6, 281.2 and 150.3 *μ*g/mL on ferrous ion chelating ability (Figure 1(c)), respectively. In addition, the total phenolic contents of crude extract, hexane fraction, EtOAc fraction, BuOH fraction, and water fraction of *A. oliverianum* leaves were 311.7, 164.1, 537.2, 457.2, and 112.3 mg GAE/g (Figure 1(d)). Accordingly, except for the ferrous ion chelating effect, the antioxidant activities of *A. oliverianum* leaves can be effectively enriched in the EtOAc fraction. It is well known that chelating agents are effective as secondary antioxidants because they reduce the redox potential, thereby stabilizing the oxidized form of the metal ion [26]. Therefore, the EtOAc soluble fraction from *A. oliverianum* leaves was not a good secondary antioxidant due to its poor capacity for metal ion binding, but it was an excellent primary antioxidant (or free radical scavenger). These results revealed that the EtOAc soluble fraction from the *A. oliverianum* leaves had a powerful antioxidant activity and it might be a good candidate to be developed as a novel natural antioxidant. Thus, the EtOAc soluble fraction was further derived into 9 subfractions by column chromatography.  Table 3 shows the elution solvent, collected weight (wt%) and DPPH radical scavenging activity for these 9 subfractions. Of these, the EA5, EA6, and EA7 exhibited the strongest inhibitory activity against DPPH radical. In addition, by HPLC separation, 8 specific antioxidants, including gallic acid (**1**), gallic acid methyl ester (**2**), 1,2,4,6-*O*-tetra-galloyl-*β*-d-glucopyranoside (**3**), 1,2,3,4,6-*O*-penta-galloyl-*β*-d-glucopyranoside (**4**), quercetin 3-*O*-*β*-d-(2′′-galloyl)-glucopyranoside (**5**), quercetin 3-*O*-*β*-d-glucopyranoside (**6**), quercetin 3-*O*-*α*-l-arabinopyranoside (**7**), and kaempferol 3-*O*-*β*-d-glucopyranoside (**8**) were further isolated from the EA 5, EA6, and EA7 (Figure 2), and their contents were determined to be 2.75 ± 0.08, 0.41 ± 0.01, 3.42 ± 0.08, 3.37 ± 0.05, 3.55 ± 0.04, 2.64 ± 0.05, 2.95 ± 0.05, and 0.73 ± 0.06 mg per gram of crude extract, respectively (Table 4). As shown in Table 4, 1,2,4,6-*O*-tetra-galloyl-*β*-d-glucopyranoside (**3**), 1,2,3,4,6-*O*-penta-galloyl-*β*-d-glucopyranoside (**4**) exhibited the strongest DPPH radical scavenging activity, and their IC_50_ values were 3.0 and 2.8 *μ*M, respectively. In addition, the decreasing superoxide radical scavenging activity order of 8 phytochemicals in NBT assay can be ranked as 4 > 5 > 3 > 1 > 6 > 7 ≫ 2 & **8**. In other words, except for compounds **2** and **8**, all the other compounds exhibited an excellent superoxide radical scavenging activity. Furthermore, the results indicated that compounds **3** and **4** (both belonging to hydrolysable tannins group) were the major bioactive phytochemicals in the extracts of *A. oliverianum* leaves. The DPPH radical and superoxide radical scavenging activities of these two phytochemicals were totally higher than those of (+)-catechin. This result also implied that the galloyl moiety played an important role for enhancing antioxidant activities. On the other hand, comparison of the antioxidant activity of flavonoid glycosides (compounds **5**–**8**), we found that quercetin 3-*O*-*β*-d-glucopyranoside (**6**) was more powerful antioxidant than kaempferol 3-*O*-*β*-d-glucopyranoside (**8**). Accordingly, it revealed that 3′,4′-dihydroxyl group on the B-ring could enhance the antioxidant activity of flavonoids. Additionally, the antioxidant activity of quercetin 3-*O*-*β*-d-(2′′-galloyl)-glucopyranoside (**5**) was better than quercetin 3-*O*-*β*-d-glucopyranoside (**6**). This result also indicated that gallate acylation on the glycoside moiety could be related to enhancing their antioxidant activity.

## 4. Conclusions

The leaf extracts of different indigenous species in Taiwan were assayed to explore their antioxidant activities. These results indicate that a number of extracts present significant antioxidant activities. Among 18 tree species, the *A. oliverianum* leaves extracts exhibited the strongest antioxidant activity, especially on the EtOAc soluble fraction, and 8 specific and excellent antioxidants were detected and identified. These results imply that the extracts or the derived phytochemicals from *A. oliverianum* leaves could be used to prevent diseases caused by the overproduction of radicals and might also be suitable for the treatment of degenerative diseases.

## Figures and Tables

**Table 1:** Antioxidant activities of methanolic extracts from the leaves of 18 indigenous tree species.

Specimens	IC_50_ (*μ*g/mL)			Total phenolic content (mg GAE/g)
	DPPH radical	Superoxide radical	Ferrous-ion chelating	
Softwood				
*C. formosana*	33.3 ± 0.2^DE^	35.8 ± 0.8^CD^	607.9 ± 8.9^C^	150.3 ± 1.3^E^
*C. japonica*	98.3 ± 2.3^A^	>100	>1000	18.9 ± 0.9^J^
*C. lanceolata *var. *lanceolata *	98.9 ± 2.4^A^	>100	297.9 ± 3.1^FG^	61.6 ± 0.4^HI^
*C. lanceolata* var. *konishii *	37.7 ± 0.6^CD^	44.2 ± 3.8^A^	439.5 ± 7.9^D^	105.2 ± 1.0^FG^
*P. morrisonicola*	25.9 ± 0.2^F^	26.1 ± 0.7^EF^	885.3 ± 10.7^A^	108.9 ± 5.3^FG^
*P. taiwanensis*	38.3 ± 1.6^CD^	38.7 ± 0.9^ABC^	>1000	120.1 ± 1.0^F^
*T. cryptomerioides*	35.9 ± 0.5^DE^	36.0 ± 1.4^BCD^	795.3 ± 11.3^B^	70.1 ± 1.5^HI^

Hardwood				
*A. confusa*	12.9 ± 0.0^GH^	22.3 ± 1.9^F^	356.7 ± 6.3^DEF^	190.2 ± 4.2^CD^
*A. oliverianum*	5.8 ± 0.1^IJ^	11.8 ± 0.6^G^	88.1 ± 1.2^JK^	311.7 ± 7.7^A^
*E. lepta*	76.7 ± 2.4^B^	>100	581.1 ± 15.2^C^	72.8 ± 0.7^H^
*F. formosana*	24.2 ± 0.4^F^	44.4 ± 1.7^A^	654.5 ± 8.7^C^	183.9 ± 1.0^D^
*H. rengetiensis*	31.6 ± 0.5^E^	42.6 ± 0.8^AB^	>1000	63.4 ± 1.0^HI^
*L. formosana*	14.9 ± 0.2^G^	30.9 ± 1.2^DE^	123.1 ± 2.0^IJ^	147.4 ± 4.8^E^
*M. kusanoi*	42.2 ± 0.7^C^	>100	185.3 ± 4.1^HI^	54.7 ± 0.3^I^
*N. konishii*	25.1 ± 0.1^F^	37.3 ± 2.5^BCD^	251.6 ± 5.1^GH^	100.0 ± 2.0^G^
*S. formosana*	38.1 ± 0.8^CD^	21.9 ± 0.8^F^	907.5 ± 69.3^A^	151.5 ± 3.4^E^
*V. luzonicum*	15.3 ± 0.2^G^	24.7 ± 0.4^EF^	316.3 ± 1.9^EFG^	210.9 ± 5.1^B^
*Z. serrata*	9.7 ± 0.2^HI^	23.6 ± 0.5^F^	397.0 ± 5.0^DE^	200.9 ± 3.4^BC^

(+)-Catechin	2.6 ± 0.0^J^	8.8 ± 0.3^G^	—	
EDTA	—	—	7.0 ± 0.4^K^	

Results are mean ± SD (*n* = 3). Different capital letters in superscript indicate significant differences among groups (*P* < 0.05).

**Table 2:** Correlation coefficients among DPPH radical scavenging activity (DPPH assay), superoxide radical scavenging activity (NBT assay), ferrous ion chelating ability (Chelating assay), and total phenolic contents (TPC) in extracts.

	DPPH assay	NBT assay	Chelating assay	TPC
DPPH assay	—	0.88**	0.37	0.71**
NBT assay	0.88**	—	0.15	0.72**
Chelating assay	0.37	0.15	—	0.54*
TPC	0.71**	0.72**	0.54*	—

*P < 0.05. **P < 0.01.

**Table 3:** Mobile phase, yields, and DPPH radical scavenging activity of EtOAc soluble fraction from the *A. oliverianum* leaves.

Subfractions	Mobile phase^a^ (v/v)	Yields (wt%)	DPPH radical inhibition (100 *μ*g/mL)	DPPH radical inhibition (10 *μ*g/mL)
EA1	5/95 (E/H)	0.9	8.2	<10
EA2	10/90 (E/H)	0.2	27.7	<10
EA3	20/80 (E/H)	0.5	27.6	<10
EA4	30/70 (E/H)	1.0	90.7	50.8
EA5	50/50 (E/H)	4.7	90.3	90.7
EA6	70/30 (E/H)	23.9	90.3	89.7
EA7	100/0 (E/H)	31.6	90.0	89.7
EA8	10/90 (M/E)	29.3	90.0	73.9
EA9	30/70 (M/E)	7.6	89.7	80.4

^a^E: Ethyl acetate; H: *n*-Hexane; M: Methanol.

**Table 4:** Antioxidant activities and contents of major phytochemicals from the* A. oliverianum* leaves.

Phytochemicals	Contents (mg/g of methanolic extract)	Free radical scavenging activity (IC_50_, *μ*M)	
		DPPH radical	Superoxide radical
**1**	2.75 ± 0.08^C^	8.2 ± 0.2^CD^	16.2 ± 1.3^B^
**2**	0.41 ± 0.01^E^	8.6 ± 0.2^C^	>50
**3**	3.42 ± 0.08^A^	3.0 ± 0.1^F^	10.3 ± 1.0^C^
**4**	3.37 ± 0.05^A^	2.8 ± 0.1^F^	6.5 ± 0.4^D^
**5**	3.55 ± 0.04^A^	5.9 ± 0.1^E^	8.6 ± 0.2^CD^
**6**	2.64 ± 0.05^C^	11.6 ± 0.4^B^	17.4 ± 0.3^B^
**7**	2.95 ± 0.05^B^	19.6 ± 0.7^A^	18.1 ± 0.7^B^
**8**	0.73 ± 0.06^D^	>50	>50
(+)-Catechin	—	7.7 ± 0.1^D^	47.1 ± 0.4^A^

Results are mean ± SD (*n* = 3). Different capital letters in superscript indicate significant differences among groups (*P* < 0.05).
